# G-InforBIO: integrated system for microbial genomics

**DOI:** 10.1186/1471-2105-7-368

**Published:** 2006-08-04

**Authors:** Naoto Tanaka, Takashi Abe, Satoru Miyazaki, Hideaki Sugawara

**Affiliations:** 1 Center for Information Biology and DDBJ, National Institute of Genetics 1111 Yata, Mishima, Shizuoka 411-8540, Japan; 2 Institute for Bioinformatics Research and Development (BIRD), Japan Science and Technology Corporation (JST), 5-3 Yonbancho, Chiyoda-ku, Tokyo 102-8666, Japan; 3 Laboratory of Information Biology, Faculty of Pharmaceutical Science, Tokyo University of Science, 2641 Yamazaki, Noda, Chiba 278-8510, Japan; 4 SOKENDAI, Hayama, Kanagawa 240-0193, Japan

## Abstract

**Background:**

Genome databases contain diverse kinds of information, including gene annotations and nucleotide and amino acid sequences. It is not easy to integrate such information for genomic study. There are few tools for integrated analyses of genomic data, therefore, we developed software that enables users to handle, manipulate, and analyze genome data with a variety of sequence analysis programs.

**Results:**

The G-InforBIO system is a novel tool for genome data management and sequence analysis. The system can import genome data encoded as eXtensible Markup Language documents as formatted text documents, including annotations and sequences, from DNA Data Bank of Japan and GenBank encoded as flat files. The genome database is constructed automatically after importing, and the database can be exported as documents formatted with eXtensible Markup Language or tab-deliminated text. Users can retrieve data from the database by keyword searches, edit annotation data of genes, and process data with G-InforBIO. In addition, information in the G-InforBIO database can be analyzed seamlessly with nine different software programs, including programs for clustering and homology analyses.

**Conclusion:**

The G-InforBIO system simplifies genome analyses by integrating several available software programs to allow efficient handling and manipulation of genome data. G-InforBIO is freely available from the download site.

## Background

The number of microbial genomes for which sequence data are available is increasing each year. Currently, complete nucleotide sequences of more than 300 strains are available in the International Nucleotide Sequence Database (INSD), which includes DDBJ, EMBL, and GenBank [1], and the sequence data are summarized in the portal site, Genome Information Broker (GIB) [2,3]. Genome data are composed primarily of annotation and sequence data, and the large volume of annotation data and long nucleotide sequences must be integrated for effective genome research. Such genome data are used for analyses that include comparisons of genomic structures between closely related species [4,5], phylogenetic analysis [6], and detection of ubiquitous [7,8] and species-specific genes (ORFans) [9,10]. It appears that genomic analyses require high-capacity computers and many programs to study multiple long sequences.

Software programs, including Artemis [11], ASAP [12,13], ERGO [14], and GenDB [15], have been developed to integrate annotation data and the results of various sequence analyses. However, a compact and easy-to-use sequence analysis package is needed by research laboratories outside of genome sequencing centers. Therefore, we developed the G-InforBIO system, an integrated system for analysis of microbial genomes that functions as a data management and sequence analysis program. Herein, we describe the functions of the G-InforBIO system and illustrate its uses with microbial genome data.

## Implementation

### System architecture

The G-InforBIO system is programmed in Java (j2sdk1.4.2_05), which is one of the most widely used computer languages in bioinformatics [16]. The inputs are annotation and whole-genome sequence files. Annotation files formatted as a flat file (FF), eXtensible Markup Language (XML), and tab-deliminated text can be imported. The genome database is constructed automatically after importing. The database items generated on the G-InforBIO system are Accession, Feature, Location, Qualifier-key, Qualifier-value, and Whole Sequence. The definitions of these items, except Accession and Whole sequence, are provided on the International Nucleotide Sequence Database Collaboration (INSDC) web site [17]. Terms in the Accession and Whole sequence fields should be unique to each genome. The Whole Sequence is the name of a whole-genome sequence file with the extension wgs, formatted as a fasta file. When an FF is imported, the whole-genome sequence file is produced automatically. In the G-InforBIO system database, annotation data are listed, and annotation data for one gene in a genome are recorded separately in multiple lines. The lines have a common term in each Accession, Location or Whole Sequence field to identify the gene location and the genome, and each of the lines has specific terms in Feature, Qualifier-key, and Qualifier-value fields respectively to represent annotation information. Lines can be extracted using keyword searches by annotation data from the database in the G-InforBIO system.

### Gene and protein sequence data retrieval from the database

Nucleotide and predicted protein sequences in the database can be retrieved for use by the analysis programs integrated in G-InforBIO. Specific nucleotide sequences can be cut out with reference to the Location field of the extracted lines in the database from the whole-genome sequence files assigned in the "Whole Sequence" field. The excised nucleotide sequences are complementary, joined, and partial sequences, whose description styles are defined in INSDC [17]. Predicted protein sequences are recorded in the Qualifier-value field, which lines have translation in the Qualifier-key field in the database. Specific predicted protein sequences encoded by the same gene locations as the extracted lines are retrieved from their predicted protein sequences recorded in the database. Sequences in a multi-fasta file are named with line numbers in the database, followed by Accession, Feature, and Location fields for each line extracted. If no lines are extracted in the database, all nucleotide or predicted protein sequences are retrieved. Retrieved nucleotide and protein sequences can be transferred to the analysis programs in G-InforBIO or be exported as a multi-fasta file. Additionally, locations selected by clicking on the database can be also retrieved and transferred to the analysis programs as the same manner.

### Sequence analysis

The G-InforBIO system contains nine programs for sequence analysis. ClustalW [18], BLASTCLUST [19], and Self-Organizing Map (SOM) [20,21] can be used for clustering analyses based on sequence similarity. BLAST [22], Blat [23], DDBJ Blast [24], MegaBLAST [25], and Sim4 [26] can be used for homology analyses, and primer3 can be used for primer design [27]. Results of some of these programs are displayed as text documents, and it may be difficult to interpret the data. Therefore, graphical result viewers were designed to display results of ClustalW [18], BLASTCLUST [19], SOM [20,21], BLAST [22], Blat [23], MegaBLAST [25], and Sim4 [26] analyses in G-InforBIO.

Furthermore, results from one analysis program can be simply utilized for the other analysis programs through a sequence file. For example, a dataset (fasta file format) of nucleotide sequence clusters generated by BLASTCLUST [19] can be imported into ClustalW [18] for phylogenetic analysis.

### Graphical genome viewer (feature Viewer)

The Feature Viewer in the G-InforBIO system can display maps of two genomes contained in the database. Gene location and annotation information are retrieved from the database. There are two Feature View windows, and each window is composed of the map viewer and the sequence viewer. In the map viewer, gene regions are represented as pentagons for genes with reference to their location information, and annotation data of genes appear in a table in this viewer. In the sequence viewer, users can browse the nucleotide sequence around a selected gene by clicking on the map viewer.

A specific nucleotide sequence selected by users can be also excised from a sequence as a text file in the Feature Viewer. Additionally, the selected nucleotide sequence is translated automatically into six-frame protein sequences. Retrieved nucleotide and protein sequences can then be captured and transferred to the analysis programs in G-InforBIO.

### Download of genomic data from DDBJ

We used the Simple Object Access Protocol (SOAP) interface [28,29] in the G-InforBIO system to download FFs with the extension .seq of genome data from the SOAP server of XML Central of DDBJ [30] in the GIB [2,3]. FFs published from GenBank, which have the extension .gbk, can be imported after they are downloaded manually from the file transfer protocol (ftp) site [31].

## Results

We used the G-InforBIO system to analyze FFs containing genomic data of two *Xylella fastidiosa *strains. It was reported that their genomic differences are limited to phage-associated chromosomal rearrangements and deletions [32], and their genome structures were compared with G-InforBIO.

### Download and import of FFs

**Figure 1:**
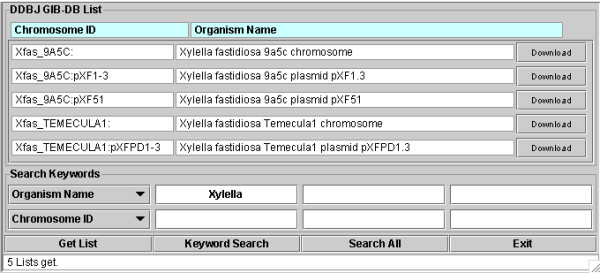
**Remote DB screen**. The list can be updated by clicking Get List, and users can obtain an FF by clicking the Download button beside the organism name in the list.

Available genomes, including chromosomes and plasmids, are listed in the Remote DB window of G-InforBIO. As shown in Fig. 1, target genomes were retrieved from the list with Xylella as keywords of Organism name, and their FFs were downloaded. The *X. fastidiosa *genome database were constructed on G-InforBIO by importing 5 FFs downloaded, including genomes of *X. fastidiosa *9a5c (1 chromosome and 2 plasmids) and *X. fastidiosa *Temecula (1 chromosome and 1 plasmid).

### Keyword searches of genes and retrieving sequences from the database

**Figure 2:**
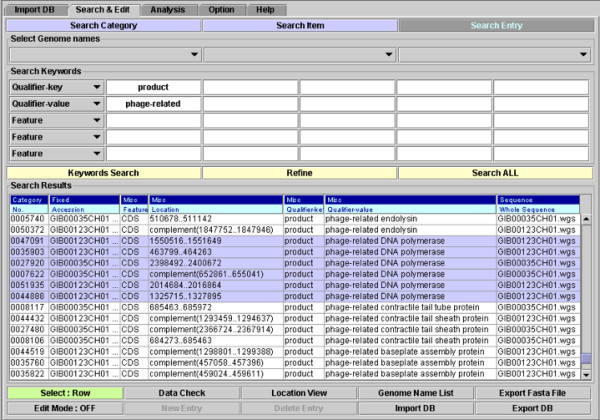
**G-InforBIO database screen**. The genome information database screen is shown. Details of the keyword searches are described in the text. Lines, including phage-related DNA polymerase, are selected by clicking in the database (as shown by violet), and their protein sequences were transferred to ClustalW as described in text.

Gene annotation information is listed in the Search Entry window, and imported genome data can be browsed. From the *X. fastidiosa *genome database, 216 phage-related genes on both *X. fastidiosa *chromosomes were extracted with keywords of product for the Qualifier-key field and phage-related for the Qualifier-value field as shown in Fig. 2. Protein sequences encoded by the 216 extracted genes were directly transferred to the analysis programs.

### Graphical viewer for comparative genomics

**Figure 3:**
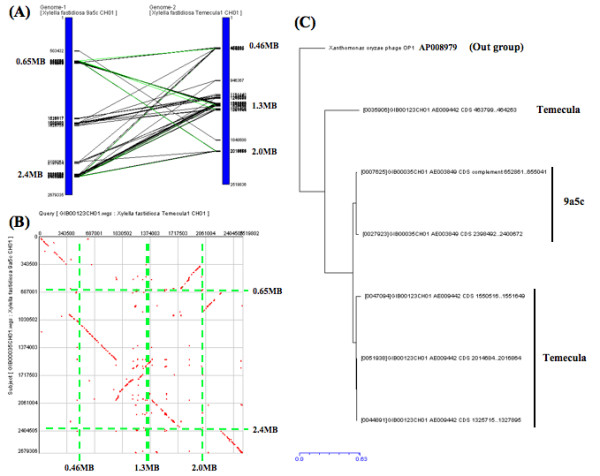
**Captures of graphical result viewers**. (A) BLSTCLUST. A protein-coding region on a genome is connected to coding regions for clustered proteins encoded on another genome with a line. User can select a protein-coding region on a genome, and lines connecting coding regions for members of a cluster including the selected protein are shown by green lines. (B) MegaBLAST. Identified common regions between two genomes are shown by a dot plot matrix. Green lines represent gene locations, which encode phage-related proteins in a cluster, generated with BLASTCLUST as described in text. (C) ClustalW. AP008979 is an accession number, issued by INSDC [17]. A scale bar indicates amino acid substitutions per position in the sequence.

The retrieved phage-related protein sequences and genome sequences of two *Xylella fastidiosa *strains, 9a5c and Temecula, were analyzed with some analysis programs integrated in G-InforBIO and compared with graphical result viewers. BLASTCLUST [19], which is based on the BLAST score-based single-linkage clustering, was used for identification of similar phage-related proteins between the two strains under the default conditions, and then 111 of 216 retrieved sequences were assigned to 36 clusters, which respectively encompassed 2 to 7 proteins. The graphical result viewer of BLASTCLUST [19], which shows the distribution of the clustered protein coding regions on both chromosomes as shown in Fig. 3A, revealed that genes for the clustered phage-related proteins are concentrated in particular locations on each chromosome. MegaBLAST [25], which is called Alignment View in the system, is used for alignment across entire genomes to identify common regions and identified it many regions common between the two chromosomes. The graphical result viewer of MegaBLAST [25], which shows the distribution of regions common between two genomes as shown in Fig. 3B, revealed fragmentations and complex inversions in the chromosome structures. Green dashed lines in Fig. 3B show locations of genes for phage-related proteins in a cluster, generated with BLASTCLUST, on each chromosome. Interestingly, the clustered phage-related proteins by BLASTCLUST were encoded near the ends of the inverted fragments and near deleted regions on each chromosome. Additionally, six phage-related DNA polymerase sequences from two strains, which are encoded near other phage-related protein genes, were retrieved as shown in Fig. 2 and were aligned by using ClustalW [18]. Their phylogenetic relationships were examined by using the neighbor-joining method [33] with the DNA polymerase sequence encoded by ORF40 on *Xanthomonas oryzae *phage OP1 genome [DDBJ: AP008979] as an out group in G-InforBIO. The capture of the result is shown in Fig. 3C. They were assigned to three clusters, which encompassed the 9a5c cluster (two phage-related DNA polymerases on 9a5c strain chromosome), the Temecula cluster (three phage-related DNA polymerases on Temecula strain chromosome), and a single cluster (one phage-related DNA polymerase on Temecula strain chromosome). The 9a5c and the Temecula clusters were closely related to each other. Thus, it seems that chromosomal rearrangements and deletions of two *Xylella fastidiosa *strains are affected by the infection of closely related bacteriophages and that Temecula strain might be additionally infected by another bacteriophage.

## Conclusion

We developed the G-InforBIO system, which allows seamless handling of genome data from management to analysis. The system is also helpful for interpretation of results because it provides a graphical view of the linkage of the data and results of various analyses. The results of analyses, however, depend on the quality of the annotation information, such as predicted coding regions, for specific genes. Genome data can be constantly updated through downloads of current data from INSDC [17] by the system.

New genome analysis tools and algorithms will be developed in the future, and the object-oriented architecture of the G-InforBIO system will allow integration of programs constructed in Java or C language. Therefore, we anticipate that this system will expand to contain additional tools for genomic analysis. The system allows comprehensive utilization of genome information. This system can be used to analyze fungal genomes in G-InforBIO.

The current G-InforBIO system can be downloaded from the download site [34], and its source code is also available as Additional file 1.

## Availability and requirements

Project name: InforBIO project;

Project homepage: ;

Operating systems: Windows 2000/XP, Macintosh OSX, Linux, UNIX;

Other requirements: CPU ≥ 1 GHz, Memory ≥ 512 MB, HD ≥ 60 MB (+ capacity for genome data), Screen resolution ≥ 800 × 600 pixels;

Programming language: Java (j2sdk1.4.2_05);

License: GNU GPL;

Any restrictions to use by non-academics: none.

## Authors' contributions

NT participated in the design and coordination of the study and drafted the manuscript. SM conceived of the study and participated in its design. TA and HS participated in the design of the study. All authors read and approved the final manuscript.

## Supplementary Material

Additional File 1 G-InforBIO_Src_V177. Zipped file of the source code of G-InforBIO system Click here for file
